# Noncanonical IFN Signaling, Steroids, and STATs: A Probable Role of V-ATPase

**DOI:** 10.1155/2019/4143604

**Published:** 2019-05-28

**Authors:** Howard M. Johnson, Ezra Noon-Song, Chulbul M. Ahmed

**Affiliations:** Department of Microbiology and Cell Science, University of Florida, Gainesville, FL, USA

## Abstract

A small group of only seven transcription factors known as STATs (signal transducer and activator of transcription) are considered to be canonical determinants of specific gene activation for a plethora of ligand/receptor systems. The activation of STATs involves a family of four tyrosine kinases called JAK kinases. JAK1 and JAK2 activate STAT1 in the cytoplasm at the heterodimeric gamma interferon (IFN*γ*) receptor, while JAK1 and TYK2 activate STAT1 and STAT2 at the type I IFN heterodimeric receptor. The same STATs and JAKs are also involved in signaling by functionally different cytokines, growth factors, and hormones. Related to this, IFN*γ*-activated STAT1 binds to the IFN*γ*-activated sequence (GAS) element, but so do other STATs that are not involved in IFN*γ* signaling. Activated JAKs such as JAK2 and TYK2 are also involved in the epigenetics of nucleosome unwrapping for exposure of DNA to transcription. Furthermore, activated JAKs and STATs appear to function coordinately for specific gene activation. These complex events have not been addressed in canonical STAT signaling. Additionally, the function of noncoding enhancer RNAs, including their role in enhancer/promoter interaction is not addressed in the canonical STAT signaling model. In this perspective, we show that JAK/STAT signaling, involving membrane receptors, is essentially a variation of cytoplasmic nuclear receptor signaling. Focusing on IFN signaling, we showed that ligand, IFN receptor, the JAKs, and the STATs all undergo endocytosis and ATP-dependent nuclear translocation to promoters of genes specifically activated by IFNs. We argue here that the vacuolar ATPase (V-ATPase) proton pump probably plays a key role in endosomal membrane crossing by IFNs for receptor cytoplasmic binding. Signaling of nuclear receptors such as those of estrogen and dihydrotestosterone provides templates for making sense of the specificity of gene activation by closely related cytokines, which has implications for lymphocyte phenotypes.

## 1. Introduction

Our understanding of signaling by cytokines such as the interferons (IFNs) at the level of gene activation is stunningly deficient in mechanisms when compared to that of nuclear receptor signaling, as seen, for example, in the case of steroids and their receptors. The canonical model of type I and type II IFN signaling is a representative in fundamentals to that of cytokine or hormone signaling by any protein or peptide signaling via the JAK/STAT pathway.

According to this model, IFN*γ* (type II IFN) signaling involves basically heterodimeric receptors IFNGR1 and IFNGR2, Janus kinases JAK1 and JAK2, and transcription factor signal transducer and activator of transcription 1*α* (STAT1*α*, reviewed in [1–3]). IFN*γ* binds to the receptors, mostly to the IFN*γ* receptor (IFNGR1), causing autophosphorylation (activation) of and binding of the JAKs to IFNGR1. Somewhere in the activation process, JAK2 moves from IFNGR2 to IFNGR1 by some unknown mechanism. Also, somewhere in the process, IFNGR1 becomes phosphorylated in the cytoplasmic domain. These events cause binding, phosphorylation, and asymmetric dimer formation of STAT1*α*. The activated STAT1*α*, via an intrinsic nuclear localization sequence (NLS), undergoes energy-dependent nuclear translocation to promoters associated with genes that are activated by IFN*γ*.

Type I IFN signaling is quite similar to that of IFN*γ*, except that there are over 16 different type I IFN subtypes, all of which bind to the same heterodimeric IFNAR1 and IFNAR2 receptor complex. They all use JAK1 and TYK2 tyrosine kinases to phosphorylate STAT1 and STAT2, which in the activated state form a heterodimer, but some are toxic (apoptotic) at high doses while others are not. The toxic type I IFNs such as human IFN*α*2 bind the receptor with a 10-fold higher binding affinity than does the nontoxic bovine IFN*τ*, yet they have the same specific antiviral activity on the same cells [4]. There is a third protein, called IFN response factor 9 (IRF9), that associates with activated STAT1 and STAT2 to form a trimeric complex called IFN-stimulated gene factor 3 (ISGF3) [1–3]. Similar to activated STAT1*α* for IFN*γ*, ISGF3 undergoes nuclear translocation, presumably via an intrinsic NLS. Also, according to the canonical model, ISGF3 is responsible for the specific activation of genes specific for type I IFNs. Thus, signal transduction via JAK/STAT does not explain the unique biological activities of different type I IFNs.

A comparison of type I IFN signaling with that of type III IFNs (IFN*λ*s) further illuminates the inadequacy of the canonical model of JAK/STAT signaling in explaining the mechanism of the specificity of cytokine signaling. Unlike type I IFNs, interleukin 10 receptor 2 (IL10R2) and IFN*λ* receptor (IFN*λ*R) form the heterodimeric receptor for IFN*λ* [5–7]. However, like type I IFNs, IFN*λ*s use JAK1 and TYK2 kinases to activate STAT1 and STAT2 for signal transduction. Although the type I IFN receptor is ubiquitous on cells, the type III receptor is cell specific, appearing in particular on epithelial cells and some other cells such as neutrophils. The induction of reactive oxygen species (ROS) in neutrophils is inhibited by IL28A type III IFN, but not by the IFN*β* type I IFN [8]. Consistent with ROS inhibition, IL28A is therapeutic in neutrophil-mediated inflammatory arthritis and colitis [8–10]. Other neutrophil functions such as phagocytosis and cytokine production are similarly affected by the two IFNs. These results beg a revisit of the conventional canonical JAK/STAT pathway as the basis for the specificity of cytokine signaling.

The specificity of IFN signaling as well as that of over 100 other different types of cytokines, growth factors, and hormones that use the canonical JAK/STAT pathway has been attributed solely to the STATs. Although there may be some overlap in their different functions, these different factors possess unique ligand specific functions at the level of the gene, cell, and organism. The problem is that there are not enough different STATs to provide a basis for the uniqueness of all these different functions as there are only seven different STATs that function mostly as homodimers [3, 11]. This means that there are cytokines that use the same STATs, but function differently. There is no evidence that a given STAT possesses functions at the level of gene activation that are unique to the activating cytokine beyond recognition of the response element [3].

The recent demonstration of activated JAK2 in the nucleus of cells by gain-of-function mutation (JAK2V617F) or by wild-type JAK2 activated by cytokines or growth factors provides profound insight into the mechanism of cytokine signaling [12]. It also challenges the canonical model of JAK/STAT signaling. In the case of a specific cytokine such as IFN*γ*, treatment of cells results in nuclear translocation of both activated JAK2 (pJAK2) and activated STAT1*α* (pSTAT1*α*) [13]. The earlier study with JAKV617F and cytokine-activated wild-type pJAK2 showed a novel and important epigenetic function of these nuclear JAKs [12]. The activated JAKs phosphorylate histone H3 at tyrosine residue 41 (Y41). Phosphorylated H3Y41, H3pY41, causes the dissociation of heterochromatin protein 1*α* (HP1*α*) from histone H3, resulting in transcription of genes repressed by HP1*α*. Activation of JAKs in the nucleus is an important epigenetic event not considered in the canonical model, but is addressed in the noncanonical model of IFN signaling later. We will first discuss briefly the nuclear receptor signaling as it is a big indicator and a guidepost for understanding cytokine signaling as well as that of other peptide/protein signaling in terms of nuclear kinases as well as the role of enhancers/enhancer noncoding RNAs (eRNAs) in such signaling.

## 2. Nuclear Receptor Signaling, Promoters, Enhancers, and Enhancer RNAs: A Comparison with Canonical IFN*γ* Signaling

As suggested, we do not have comparable understanding of mechanisms of specific gene activation between nuclear receptor systems such as steroid/steroid receptor and canonical JAK/STAT signaling as exemplified by IFN*γ* and its receptor. A comparison of the two at the level of the promoter and enhancer region of genes that are activated by steroids versus those activated by IFN*γ* readily illustrates the difference in knowledge. In canonical signaling, IFN*γ* binds to its heterodimeric receptor subunits IFNGR1 and IFNGR2, with IFNGR1 playing a dominant role in the binding [2, 14]. JAK1 is present on IFNGR1, and the binding results in movement of JAK2 from IFNGR2 to IFNGR1. These events result in JAK autophosphorylation, phosphorylation of IFNGR1, and binding and phosphorylation of STAT1*α*, which forms an asymmetric homodimer and undergoes active nuclear transport to the promoters of genes that are activated by IFN*γ*. The canonical model does not provide insight into the movement of JAK2 from IFNGR2 to IFNGR1. Importantly, it does not show the connection between activated STAT1*α* at promoters of genes that are activated by IFN*γ* and specific IFN*γ* function as many cytokines with their own unique functions also activate STAT1*α* [11]. Next-generation sequencing (NGS) studies provide important insight in genome-wide activity of cytokines, but the problem of specificity as per IFN*γ* above is not addressed [3]. NGS also does not connect the dots of activated JAK2 in the nucleus along with activated STAT1*α* in terms of their coordinated function as activated JAK2 in the nucleus is not considered in the NGS studies.

We have previously presented an overview of nuclear receptor signaling as per steroid hormone (SH)/steroid receptor (SR) signaling [15]. We briefly revisit this and then show how mechanistic events are embedded into nuclear receptor (NR) signaling beyond promoters and extend to providing insight into the role of enhancers and long-noncoding RNAs called enhancer RNAs (eRNAs) in steroid signaling. We will then show why this is important, via noncanonical IFN signaling, in understanding signaling by cytokines like IFN*γ* not only at promoters, but also at enhancers and eRNAs.

In a nutshell, SH/SR signaling proceeds as follows. There are many reviews on the subject with considerable detail, but this overview makes our point [16]. SH binds to SR in the cytoplasm and/or the nucleus at hormone response promoter elements (HRE). SR is a transcription factor. Scaffolding proteins called steroid coactivators (SRC) bind to SH/SR through their LXXLL motifs, of which there are three [16]. They do not bind DNA directly. SRCs recruit secondary coactivators such as histone acetyltransferase p300/CBP, methyltransferases such as PRMT1 and CARM1, and chromatin remodeling complex SWT/SNF. Serine/threonine and tyrosine kinases also become a part of this machinery [16, 17]. The SH/SR complex at the promoter is a key to the mechanistic insight of gene activation, including key epigenetic events. The canonical model of IFN*γ* signaling by contrast, with only STAT at the promoter, tells us very little and is even primitive relative to that of SH/SR signaling.

The abundance of genetic and epigenetic mechanisms in SH/SR signaling as well as in other NR signaling at the promoters of genes activated by NRs provides a picture that contrasts not only with IFN*γ* signaling but also in general with peptide and protein ligands of hormone, growth factor, and cytokine signaling. The role of enhancers in gene activation is vague and even unknown for almost all ligand/receptor systems except for the case of NRs. Most of the transcribed RNA in the genome does not result in protein production, but rather plays a regulatory role in gene regulation. Of particular interest in the context of enhancers is a subgroup of long-noncoding RNAs (lncRNAs) called enhancer RNAs (eRNAs) [18–20]. eRNAs are transcribed from enhancers, and SH/SR and other NR signaling provide important insight into how eRNAs are transcribed and how enhancers interact with promoters and the role of eRNAs in this interaction [21, 22].

Chromatin immunoprecipitation (ChIP) has been applied to NGS procedures such as ChIP-seq to demonstrate that the estrogen receptor (ER) binds to 5,000 to 10,000 locations across the genome [23]. Another powerful weapon in the toolbox of NGS goes by the acronym of GRO-seq, which stands for global run-on sequencing [24]. GRO-seq is a direct, high-throughpout sequencing procedure for finding RNAs and is adapted from conventional nuclear run-on methodologies. These technologies provide insight into the binding of ER and androgen receptor (AR) to enhancers and subsequent transcription of eRNAs and interaction or cross talk with ER and AR promoters.

Active enhancers are known by the company they keep. Thus, high levels of histone 3 lysine 4 monomethylation (H3K4me), low levels of H3K4 trimethylation (H3K4me3), and increased H3K27 acetylation (H3K27Ac) are associated with active enhancers [25]. These types of alterations in H3 along with other epigenetic signals such as tyrosine kinase activity result in the exposure/unwrapping of the DNA for transcription [26]. Accordingly, these epigenetic modifications usually precede transcription of the noncoding eRNA from the enhancer DNA. The transcription of eRNA is carried out by enhancer-associated RNA polymerase II (Pol II) [25].

In the case of human breast cancer cells, treatment with 17*β*-estradiol (E2) results in E2-bound estrogen receptor *α* (ER*α*) association with enhancers adjacent to genes that are upregulated by E2 [22]. E2/ER*α* is thus bound to both the enhancers and promoters of genes that are activated by ER*α*. Similar to the ER*α* results, studies with dihydrotestosterone- (DHT-) treated prostate cancer cells also showed AR association with an enhancer involved in AR activation of specific genes [21]. ChIP assay on DHT-treated LNCaP prostate cancer cells showed a dynamic interaction of AR with the promoter and enhancer, where AR loaded onto enhancers to a greater extent, but enhancer association was more transient than that to the promoter [21]. Further, studies showed that eRNA may function as a scaffold that guides an AR-linked protein complex to target chromatin so that DHT-stimulated transcription either occurs intrachromosomally (cis activity) or interchromosomally (trans activity), depending on the promoter target of eRNA. Importantly, this finding ascribes a key function to eRNA as a bridge between the enhancer and promoter. Using steady-state histone acetyltransferase (HAT) assays, it has recently been shown that eRNA binds directly to CREB-binding protein (CBP) to enhance the HAT activity of CBP at enhancers [27]. The interaction increases CBP HAT activity by increasing its binding to histone. These studies provide insight into how eRNAs function in gene activation.

**Figure 1:**
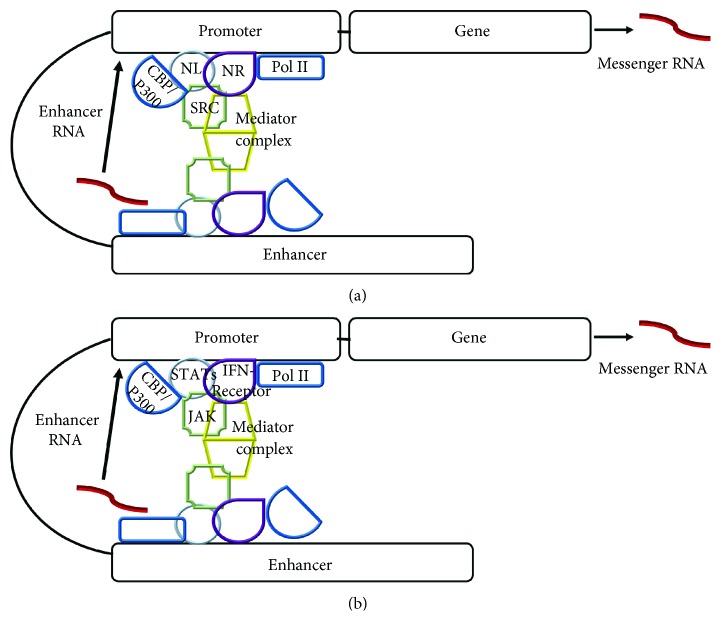
Nuclear receptor signaling as the template for IFN signaling via the JAK/STAT pathway. (a) Steroid hormones such as estrogen and dihydrotestosterone nuclear receptor ligands (NL) signal through cytoplasmic soluble nuclear receptors (NRs). NR signaling involves scaffolding proteins called steroid receptor coactivators (SRC) which serve as the platform for other factors such as histone acetyltransferases (CBP/p300) and kinases as well as other players such as a mediator complex consisting of 26 or more protein subunits. These factors are present at both the promoter and enhancer of genes specifically activated by steroids and their receptors. Specific transcription occurs at both the promoter (mRNA) and enhancer (eRNA) by RNA polymerase II. (b) We have shown that interferon (IFN) signaling involves the presence of IFN*γ*, IFN*γ* receptor, activated JAKs, activated STATs, and transferases at the promoters of genes activated by IFNs. It is predicted that similar players are present at the relevant enhancer of genes activated by IFNs, a prediction that can readily be tested. Adherents of the canonical model of IFN signaling do not conceptually accept retrograde trafficking of plasma membrane receptors to the nucleus, although this has been widely shown in JAK/STAT signaling as well as in receptor tyrosine kinase signaling (see text).

A current picture of the players at the enhancers and at the promoters of genes activated by steroid hormones such as estrogen and testosterone is presented in Figure 1(a). For both the promoter and enhancer, there are a collection of similar players. Nuclear receptors such as ER and AR with ligand attached function as transcription factors at both the promoter and enhancer. Thus, at the enhancer, eRNA is transcribed, while at the promoter, messenger RNA (mRNA) is transcribed. The SRC cofactors and platforms are present at both sites as well as are the epigenetic factors such as CBP/300. Pol II catalyzes the synthesis of both eRNA and mRNA, and eRNA synthesis is bidirectional. The mediator complex in Figure 1(a) consists of 26 or more subunits in mammals and plays a key role in Pol II activity, such as preinitiation, initiation, reinitiation, pausing, and elongation [28]. Topoisomerase I is recruited to AR-bound enhancers in order to nick DNA to relieve supercoiling and allow DHT-regulated eRNA synthesis to occur [29]. The looping shown in Figure 1(a) brings the enhancer complex into close proximity to the promoter for coordinated activity. There are other players in these enhancer-promoter complexes, but the foundation factors for specific gene activation are typified by E2 or DHT steroid ligands bound to their respective ER or AR nuclear receptor. Specific gene activation does not occur without them. Cytokine, growth factor, and polypeptide (or protein) ligand signaling does not entertain many of these steroid signaling counterparts and their receptors in complexes such as in Figure 1(a). We show with particular focus on IFN below that this hinders access to specific mechanisms in signaling by these factors.

## 3. The Foundations of the Steroid-Like Noncanonical IFN Signaling Model

Structural studies of protein/peptide ligands binding to the membrane receptor extracellular domain are generally looked upon as key to gaining mechanistic insight to signaling that occurs at the cytoplasmic domain of the receptor. In the case of IFN*γ*, details of IFN*γ* interaction with the IFNGR1 receptor subunit were obtained using X-ray crystallography [30]. IFNGR1 plays the key role in IFN*γ* binding to receptors in intact cells. The structural studies focused on interaction between IFN*γ* and IFNGR1 extracellular domain via well-defined secondary structures. The C-terminus of IFN*γ*, which contains a polycationic tail, did not form a clearly defined secondary structure and did not show interaction with the IFNGR1 extracellular domain. Interestingly, the C-terminus polycationic tail functions as a classic nuclear localization sequence (NLS) [31]. To date, this fact has not received much attention by the adherents of the canonical signaling pathway of IFNs even though the NLS is required for IFN*γ* function [32].

In our early studies of binding sites on IFN*γ* for the IFN*γ* receptor in intact cells, IFN*γ* N-terminus peptide IFN*γ* (1-39), but not the NLS-containing C-terminus peptide IFN*γ* (95–132), inhibited IFN*γ* binding to the receptor on cells [33]. This was unexpected as antibodies to both the N-terminus and C-terminus peptides had similar neutralizing effects on IFN*γ*. We next used a full-length IFNGR1 soluble receptor and carried out bindings with IFN*γ* and with overlapping peptides, including IFN*γ* (1-39) and IFN*γ* (95–132). We also used overlapping IFNGR1 extracellular and cytoplasmic domain peptides. We discovered that the N-terminus peptide of IFN*γ* bound to the IFNGR1 extracellular domain and that the C-terminus peptide bound to the IFNGR1 cytoplasmic domain [33]. Specifically, murine IFN*γ* (95–132), as well as the human counterpart peptide, bound to the IFNGR1 cytoplasmic domain region (253–287), adjacent to the binding site of activated JAK2. The binding was specifically blocked by anti-(253–287)-specific antibodies in fixed, permeabilized cells. Related to this, the binding of Sepharose-coupled JAK2 to labeled soluble IFNGR1 was enhanced by IFN*γ* and IFN*γ* (95–132), but not by the N-terminus IFN*γ* (1-39) peptide. Binding was specifically blocked by IFNGR1-binding site peptide IFNGR1(253-287). Such enhanced binding could explain the movement of JAK2 from receptor subunit IFNGR2 to the receptor subunit IFNGR1.

The challenge was to show functional and physical evidence of intracellular IFN*γ* and IFN*γ* (95–132) in treated cells. The first step was to show that internalized IFN*γ* (95–132) possessed IFN activity. Thus, macrophages with active pinocytosis internalized IFN*γ* (95–132) and induced antiviral activity as well as upregulation of MHC class II antigens [34]. Attachment of a palmitate residue to IFN*γ* (95–132), Pal-IFN*γ* (95–132), for internalization by fibroblasts, similarly resulted in the induction of antiviral activity and upregulation of MHC class II antigens [35]. Knockout of the IFNGR1 gene in fibroblasts resulted in loss of IFN*γ* peptide Pal-IFN*γ* (95–132) function which is evidence that the C-terminus peptide functions through IFNGR1.

There has long been evidence that internalized IFN*γ* also possesses biological activity across species. For example, the following have been reported: (1) liposome-encapsulated human IFN*γ* induced an antitumor effect in murine macrophages [36], (2) intracellular human IFN*γ* induced an antitumor effect in murine fibroblast cells [37], and (3) human IFN*γ* microinjected into murine macrophages induced MHC class II antigen expression in murine macrophages [38]. These intracellular effects of human IFN*γ* are odd for two reasons. First, protein ligands like IFN*γ* are supposed to function by binding to extracellular receptor domains [1, 2]. Second, human IFN*γ* does not have a biological effect on murine cells when simply added to these cells in cultures, because the murine IFN*γ* receptor extracellular domain does not recognize human IFN*γ* [39]. These cross-species intracellular effects of human IFN*γ* have had to sit in limbo, waiting for a mechanism of IFN signaling that did not exist at the time of these discoveries.

Considerable insight has been gleaned concerning trafficking of the IFN*γ* ligand and IFNGR1 receptor subunit from the plasma membrane to the nucleus. Specifically, we showed that endocytosed IFN*γ* associates with the cytoplasmic domain of the receptor IFNGR1 subunit in the following manner [32]. Unlabeled IFN*γ* but not IFNGR1(253-287) intracellular binding site peptide blocked binding of ^125^I-IFN*γ* to the IFNGR1 extracellular domain. Internalized IFNGR1(253-287), however, blocked intracellular cytoplasmic binding of ^125^I-IFN*γ* to IFNGR1 subsequent to extracellular binding. In the determination of internalization dynamics of IFN*γ* receptors, we showed that the presence of IFNGR1 and IFNGR2 in the lipid microdomain on the surface of the cell was central to the endocytic events that are linked to the IFN*γ* noncanonical signaling pathway [40]. In human epithelial WISH cells, the receptor subunits IFNGR1 and IFNGR2 are constitutively present in lipid microdomains, while in Jurkat cells, the receptor subunits migrate to the lipid microdomain. While IFNGR1 undergoes nuclear translocation in cells treated with IFN*γ*, receptor subunit IFNGR2 remains in the plasma membrane [40, 41]. This raises questions about how the receptor subunits are cross-linked by IFN*γ* or if cross-linking in fact occurs in a IFNGR1/IFNGR2 manner [42, 43]. The cytoplasmic domain of IFNGR1 with IFN*γ* attached is exposed to the cytoplasm in endocytic vesicles, since the microinjection of antibodies to IFN*γ* C-terminus in cells blocked IFNGR1 nuclear translocation as well as STAT1*α* activation [44]. The antibodies had no effect on IFN*α* activation of STAT1*α*.

**Figure 2:**
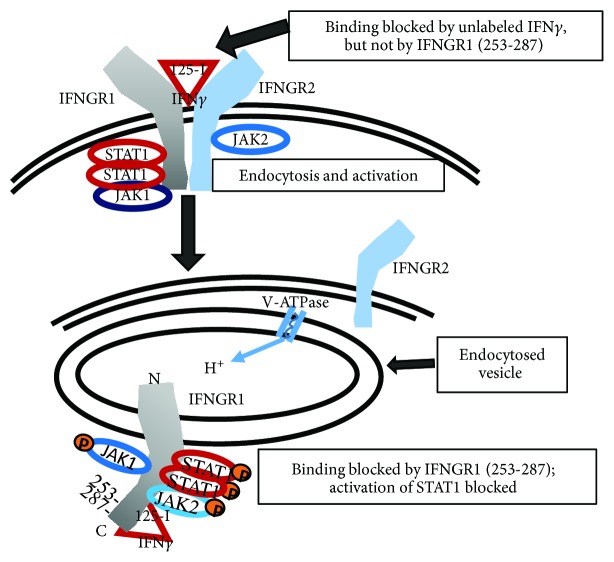
Schematic of IFN*γ* binding to the receptor subunit IFNGR1 extracellular domain with endocytosis of the complex and subsequent movement of IFN*γ* to the cytoplasmic domain of IFNGR1 at a site encompassing residues 253 to 287. Endosome-associated V-ATPase proton pump lowers the lumen pH for IFN*γ* movement to the cytosol. Accordingly, extracellular binding of ^125^I–IFN*γ* is blocked by unlabeled IFN*γ* but not peptide IFNGR1(253-287). Cytoplasmic binding of ^125^I–IFN*γ* is blocked by IFNGR1(253-287). Details of the experiment illustrated in this schematic are contained in the text [32]. Similar movement for ligands of RTKs from the receptor extracellular domain to the cytoplasm of the cell has been shown [15].

The question arises as to whether there are existing mechanisms to explain the dissociation of IFN*γ* from the IFNGR1 extracellular domain in the lumen of the endosome and subsequent association with the IFNGR1 cytoplasmic domain in the cytosol of the cell. The mechanism of these events are illustrated in Figure 2. As indicated, we showed that it is the endocytosis of IFN*γ* receptors present in lipid microdomains that are key to our noncanonical model of signal transduction [40]. Lipid microdomains and caveolae are rich in proton pumps known as vacuolar H^+^-ATPases or V-ATPases (reviewed in [45–47]). These pumps are central to pH control in organelles such as endosomes and are, in fact, a key to signal transduction associated with endocytosis. V-ATPases are structurally and functionally related to F-ATPases which are well known in synthesis of ATP against a proton gradient in mitochondria. Unlike F-ATPases, V-ATPases do not synthesize ATP under physiological conditions but rather are ATP-driven proton pumps [45]. Thus, V-ATPases associated with the endosome lower the pH in the lumen to about 4.5 [48]. This results in dissociation of complexes such as IFN*γ*/IFNGR1 [48, 49]. The cationic NLS tail of IFN*γ* becomes protonated in the low pH which facilitates crossing of the endosome membrane into the cytosol. In the cytosol, the pH is 7 and above [48], and the IFN*γ* then binds to the IFNGR1 cytoplasmic domain at residues 253-287 [33]. The experimental support for these events is as follows. The cationic NLS is very similar for IFN*γ* and the prototypical NLS of SV40 large T antigen [49]. We showed that an IFN*γ* mutant that lacked the cationic NLS was inactive and that the SV40 NLS restored complete antiviral activity [49]. In studies where SV40 NLS was coupled to an siRNA for nuclear transport of the RNA, it was shown that V-ATPase acidification of lipid membrane-derived endosomes resulted in SV40 NLS penetration of the endosome membrane and movement from the lumen side to the cytosol [50, 51]. Thus, the data of reference [32] and Figure 2 are consistent with well-known endocytic events involving V-ATPase and acidification of endocytic endosomes.

Receptor tyrosine kinases (RTKs), such as epidermal growth factor receptor (EGFR), have also been shown by different laboratories to undergo energy-dependent nuclear translocation [52, 53]. In fact, EGFR was the first RTK as well as the first plasma membrane receptor to be shown to function as a cotranscription factor [54]. The mechanism of retrograde trafficking of EGFR from the cell surface into the nucleus has been extensively elucidated [55, 56]. Endocytosis is triggered by binding of EGF to EGFR, with fusion of the endocytic vesicles with early endosomes, which then traffic to Golgi. Trafficking was blocked by treatment with either brefeldin A or by use of cells with dominant negative mutation of the small GTPase ARF (ADP-ribosylation factor). Both treatments resulted in coat protein complex I (COPI) disassembly, which is consistent with COPI regulation of retrograde vesicular trafficking of EGFR from the Golgi to the endoplasmic reticulum [55]. EGFR movement from the ER into the nucleus was shown to require the Sec 61 translocon [56]. Epigenetic changes such as tyrosine phosphorylation of histone H4 at residue H4Y72, which is associated with enhanced methylation at H4K20, accompanied nuclear translocation of EGFR [57]. Nuclear translocation and epigenetic effects have also been reported for other RTKs [58, 59].

## 4. Noncanonical IFN*γ* Signaling at the Promoter

The data presented here show a remarkable similarity between IFN*γ* noncanonical signaling and that of NRs and their ligands. The combination of immunoprecipitation with Western blotting, ChIP assay followed by PCR, nuclear confocal immunofluorescence, and other focused techniques showed that IFN*γ*, STAT*α*, JAK1, and JAK2 were all present at the GAS element of genes activated by IFN*γ* [13, 15, 44, 60]. Initial focus was on the role of IFN*γ* NLS in translocation of IFNGR1 into the nucleus. Thus, treatment of cells with IFN*γ* or with the internalizable C-terminus peptide Pal-IFN*γ* (95-132) resulted in IFNGR1 translocation to the nucleus with the NLS of IFN*γ* playing a key role for importin *α*/*β* and nuclear pore complex recognition [60]. ChIP assay has been particularly useful in showing that activated STAT1*α*, pSTAT1*α*, and activated JAKs, pJAK1 and pJAK2, form a complex with IFN*γ* and IFNGR1 at or in the vicinity of promoters of genes that are activated by IFN*γ* [13, 60].

There is evidence that IFNGR1 plays a role in gene activation by IFN*γ*. GAS-luciferase reporter gene transfection, along with IFNGR1 and nonsecreted IFN*γ*, resulted in enhanced reporter activity. Further, fusion of IFNGR1 to yeast GAL-4 DNA-binding domain resulted in enhanced transcription from the GAL4 response element, consistent with a transactivation domain in IFNGR1 [60]. These results suggest a transcriptional/cotranscriptional role for IFN*γ*/IFNGR1 in specific gene activation by IFN*γ*. In NR signaling, the factors at the promoter seem also to be present at the enhancer and we feel that IFN*γ*, IFNGR1, and pJAK2 should be included in specific gene studies as well as in genome-wide studies.

Within the IFN family, noncanonical signaling is not limited to IFN*γ*, but was also found in the type I IFN system [61]. Cells treated with IFN*α* under the same protocol conditions as for IFN*γ* showed IFN*α*2, receptor subunits IFNAR1 and IFNAR2, and the type I IFN tyrosine kinase TYK2, all at the response element ISRE of the oligoadenylate synthetase (OAS) promoter [61]. An unrelated gene promoter for *β*-actin did not show these IFN players in the same cells treated with IFN*α*2. Similarities between noncanonical IFN signaling and steroid signaling are presented in Figures 1(a) and 1(b).

As indicated, it has been shown that both wild-type and gain-of-function mutated JAK2 perform key epigenetic functions in the nucleus [12]. Mutated JAK2, JAK2V617F, plays a key epigenetic role in leukemias involving the erythropoietin (EpoR), thrombopoietin, and granulocyte colony stimulator receptors [62]. The key epigenetic effect of JAK2V617F was to phosphorylate H3 at tyrosine (Y) 41, H3Y41 to H3pY41, which results in dissociation of the inhibitor protein HP1*α* from H3. This, in conjunction with other epigenetic effects (see below) on H3, plays a key role in euchromatin activity related to gene expression in the associated cancers [12]. It has been shown that in the case of Epo, the EpoR is required for the activation of JAK2V617F [62]. The question arises as to how EpoR activated JAKV617F then goes on to play a key role in leukemias that possess an EpoR phenotype. Thus, it would be interesting to determine if EpoR-coupled JAK2V617F is the activator of Epo genes rather than just JAK2V617F alone. There is a Drosophila HP1 and mutant JAK counterpart of JAK2V617F [63].

Essentially lost in the JAK2V617F discovery is the fact that wild-type JAK2, as indicated, also phosphorylated H3 at Y41 (H3pY41) with the same epigenetic result [12]. Activation of the wild-type JAK2 was dependent on treatment of cells with growth factors like platelet-derived growth factor (PDGF) and interleukin 3 (IL-3).

ChIP-seq has recently been used to study the genomic effects of nuclear JAK1 in an autocrine IL-6 and IL-10 activated B-cell lymphoma [64]. Specifically, it was shown that JAK1 regulated the expression of almost 3,000 genes in a B-cell lymphoma cell line (ABC DLBCL), with the interesting finding that many of these same genes showed phosphorylation of tyrosine 41 at histone H3, H3pY41, in the surrounding chromatin. A JAK1 inhibitor blocked the phosphorylation. Possible association of JAK1 and IL-6-related STAT3 at the genome level was not determined by ChiP-seq, but focus on the MYC gene did show H3pY41 and pSTAT at the MYC gene as determined by quantitative ChIP analysis. The question is as follows: are they physically linked, and if so, were there IL-6 receptor players present?

An important functional role of the induction of H3pY41 by the kinase activity of JAK2 and TYK of the IFNs, as discussed here, would be evidence of related nucleosome unwrapping, so that the IFN/receptor complexes have access to the DNA. In this regard, it has been shown that JAK2 induction of H3pY41 increased nucleosome unwrapping for access to transcription factor binding by several folds [26]. Lysine 56 (H3K56) is located in the same DNA interface as H3Y41. Thus, acetylated H3K56 (H3K56ac) also caused nucleosome unwrapping by several folds. The combination of H3pY41 and H3K56ac had a multiplicative effect with 17-fold unwrapping of the nucleosome. The authors concluded that it was the combination of these two epigenetic events that resulted in optimal DNA exposure to transcription factors. The nucleosome unwrapping mechanisms described here probably apply to both promoters and enhancers and are revisited in that context in the next section.

Changes in H3K9 have also been shown to be associated with gene activation [13]. For example, we observed in type I IFN-treated cells that trimethylated H3K9, H3K9me3, underwent demethylation in association with acetylation of the same residue (H3k9ac) at the region of the OAS1 promoter [13]. Tyrosine phosphorylation of H3, H3pY41, was observed in the same experiment. All of these events show a remarkable similarity to that of NR signaling as presented above.

## 5. IFNs, Enhancers, and eRNAs

The players at the promoters and enhancers of NR signaling as presented in the previous section on signaling by E2/ER*α* and DHT/AR paint a picture that is visual and communicative. Promoters contain NR, SRCs, and secondary cofactors like p300/CBP HATs, kinases, and Pol II for transcription (reviewed in [25, 65, 66]). It is important to note that enhancers also contain many of these players, in particular NRs and their ligands, as well as a mediator complex [25, 28, 65, 66]. All of these factors play a key role in NR-specific enhancer transcription of eRNA, which in turn plays a role in enhancer/promoter interaction for NR-specific transcription of mRNA by Pol II.

Genome-wide NGS with the primary focus on STATs and superenhancer (SE) markers, such as p300 HAT and H3K4me1, showed different patterns for T helper1 (Th1) versus Th2 cells [3, 67]. There are also lineage-determining transcription factors present at SEs adjacent to promoters of genes whose products define the different T cell phenotypes, for example, T-BET/STAT1/STAT4 at relevant SEs in Th1 cells, GATA3/STAT6 at the key SEs for Th2 cells, and ROR-*γ*t/STAT3 at Th17 cell SEs [67]. The potential role of eRNAs in the determination of phenotypes was not addressed. The precise role of the lineage-determining T cell phenotype transcription factors in T cell differentiation is not clear and may be to function as negative regulators of phenotypes that differ from the signature phenotypes that they are associated with [67].

It appears that the cytokines may play a major role as inducers and possibly direct participants of signal-dependent transcription at the enhancers that participate in phenotype-specific gene activation. With regard to STATs and Th1 and Th2 cells and the respective roles of STAT4 (Th1) and STAT6 (Th2), recruitment of p300 and other enhancer signals have been attributed to these STATs based on the absence of p300 at enhancers where STATs are deficient [68]. Master regulators such as T-bet do not restore enhancer activity that is lost as a result of the STAT deficiency. As we indicated earlier, there is a problem in assigning specificity solely to STATs in cytokine signaling at the level of enhancers and promoters (reviewed in [3]). For example, IFN*γ* treatment of cells results in a dramatic increase in STAT1 at putative enhancers of IL-6 and tumor necrosis factor [3]. Interaction of STATs with enhancers and other noncoding DNA sites is greater in terms of binding than with promoters as there is more of the noncoding DNA [3]. In some respects, rather than solving the problem of cytokine specificity regarding STATs at promoters, it is just been moved on to include enhancers.

All STATs bind to the GAS element, but how they recognize enhancer DNA is not clear. Cytokines that preferentially activate particular STATs may use another STAT or STATs if there is a deficiency of the preferred STAT. For example, STAT3 may substitute for STAT1 or STAT6 may substitute for STAT5 [3]. Different STATs may congregate at the same DNA elements and/or interact with other transcription factors and even compete with each other for the same DNA element [3]. Presumably, this also applies to enhancers. Additionally, cytokines may preferentially activate a particular STAT but also activate to a lesser extent the other STATs [3]. These and other complex STAT behaviors have recently been dubbed “the STAT specificity paradox” [3].

We are not aware that any of the extensive NGS studies with STATs has differentiated between activated (phosphorylated) or nonactivated (unphosphorylated) STATs at enhancers or at other regions of the genome. Activated STATs have been reported to be associated with euchromatin, while nonactivated STATs were associated with heterochromatin [63, 69]. Euchromatin indicates active DNA while heterochromatin indicates silent DNA. We thus feel that the discovery of cytokine and growth factor treatment of cells, where activated JAK2 underwent nuclear translocation, has profound importance for epigenetic activity of the genome. We have addressed earlier the histone H3Y41 to H3pY41 phosphorylation, which results in dissociation of the epigenetic inhibitor protein HP1*α* from histone H3, resulting in unwrapping of the nucleosome and exposure of the DNA, presumably at both the promoter and enhancer [12, 26]. We determined that in cells treated with IFN*γ*, activated JAK2 (pJAK2) underwent nuclear translocation along with activated STAT1*α* (pSTAT1*α*) [13, 70]. Similarly, when we treated cells with IFN*α*, activated TYK2 (pTYK2) underwent nuclear translocation [61]. In both cases, activated STAT1 also underwent nuclear translocation to the same promoters in a complex that contained the activated JAKs. The presence of pJAKs and pSTAT1 at the same enhancers was not determined but is likely very important for enhancer function. The fact that a particular cytokine/receptor interaction results in nuclear import of pJAKs and pSTATs intuitively tells us that there is probably cross talk between the two at enhancers and promoters of genes that are activated by the particular cytokine. The data in Section 4 is based on our finding that IFN*γ* and receptor subunit IFNGR1 are part of these nuclear events involving pJAK2 and pSTAT1*α*, analogous to NR signaling, which could help resolve “the STAT specificity paradox” [3].

One does not need to fully subscribe to our NR-related noncanonical model of IFN signaling in order to have interest in associations of STATs at promoters and enhancers. We do not yet know if STATs bind to eRNAs, but there is a report of a lncRNA that is involved in conventional dendritic cell (cDC) differentiation which associates with and plays a role in STAT3 phosphorylation in the cytoplasm [71]. Nuclear colocalization of the two was not observed, which suggested that the lncRNA function was restricted to the cytoplasm. A series of experiments showed that the lncRNA protected pSTAT3 from the protein tyrosine phosphatase SHP1. Knockdown of lncRNA promoted the association of SHP1 with pSTAT3. An lncRNA associated with influenza virus infection called negative regulator of antiviral response (NRAV) was downregulated in virus-infected cells, which was associated with the suppression of IFN-stimulated gene transcription [72]. In terms of players at enhancers and promoters, STATs have been shown to associate with the HAT p300. A HAT CBP/p300 has recently been shown to associate with eRNA in RNA-dependent acetylation such as H3K27ac [27]. All of this suggests that STATs, presumably at enhancers, bind to CBP/p300, which in turn has been shown to bind to eRNA. How, then, are STATs associated with eRNAs?

It should be noted that CBP/p300 is highly prone to interactions with intrinsically disordered proteins (IDPs) as well as with proteins with intrinsically disordered regions (IDRs) [73]. IDPs and IDRs are particularly abundant in eukaryotic transcription factors. STATs and NF-kappaB as well as CBP/p300 are all IDPs and/or IDR proteins [73]. IFN*γ* has a C-terminus that qualifies as an IDR [30]. It has been proposed that IDPs have functional advantages in the mediation of transcriptional events involving small recognition motifs with flexibility for multiple target interactions, thus giving rise to efficient use of small binding regions, as well as possessing the ability for high specificity with modest affinity for ease of reversible interactions [73]. The proteins in our noncanonical IFN signaling model from the ligand and receptor to genetic and epigenetic proteins at promoters and enhancers all qualify as IDPs or IDR proteins. It seems that those fuzzy nonstructured regions of many impressive structural studies may contain more than what meets the eye.

**Figure 3:**
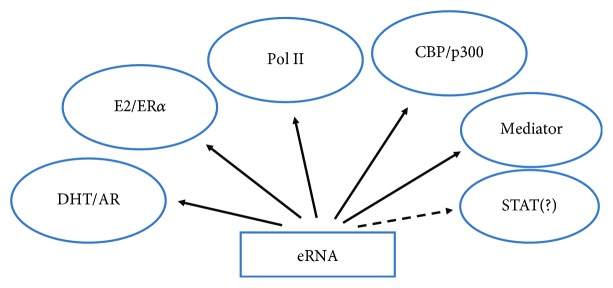
Binding of eRNA to various players in gene activation provides insight into its function. DHT/AR and E2/ER*α* binding places eRNA at the enhancer and promoter, as these nuclear receptor systems have been shown to be present at both sites. CBP/p300 histone acetyl transferase interaction with eRNA suggests epigenetic functions, while RNA polymerase II (Pol II) is involved in transcription at both the promoter and enhancer. STAT association with eRNA has not been definitively determined yet. Determination of complexes at the promoter and enhancer is key to understanding which factors directly bind to eRNA and which bind indirectly as members of transcriptional complexes. Nuclear receptor studies suggest that eRNA plays a role in looping of the enhancer and promoter via the interactions such as those shown here.

Figure 3 summarizes some of the proteins that bind to eRNA as presented in the text. There is evidence that in the case of nuclear receptor systems, such as E2/ER*α* and DHT/AR, eRNA binds to these receptors as well as to complexes of the mediator, CBP/p300, various other transcription factors, and other players at the enhancer and promoter [21, 22, 25, 27]. These interactions are thought to facilitate the looping that brings the distal enhancer and promoter into close proximity for specific gene transcription.

## 6. Conclusion

The heart and soul of studies of ligand/receptor signaling is elucidation of the mechanism of specific gene activation. For the case of NRs, the foundations are in place with the NR and ligand at promoters and enhancers of genes specifically activated by the ligand. For example, E2 and ER*α* orchestrate the transcription of eRNA at the enhancers. The eRNA appears to be involved in mRNA transcription at the promoter, which also contains E2 and ER*α*. Other factors comprise the supporting cast. In canonical JAK/STAT signaling, STAT is the counterpart of the NR and we think that herein is the problem with the interpretation of NGS studies with STATs across the genome that has resulted in “the STAT specificity paradox.” For IFN*γ* signaling, the inclusion of IFN*γ*, IFNGR1, and activated JAKs in NGS studies in the context of the noncanonical model could potentially help resolve the paradox.
